# A scientometric analysis of global research on gut microbiota and glioma

**DOI:** 10.3389/fonc.2025.1646187

**Published:** 2025-10-07

**Authors:** Lize Chen, Kun Shu, Zhiyin Zhang, Jiao Zhao

**Affiliations:** Department of Neurosurgery, Northwest University First Hospital, Xi’an, Shaanxi, China

**Keywords:** glioma, gut microbiota, bibliometrics, VOSviewer, CiteSpace

## Abstract

**Background:**

Most previous studies have demonstrated that gut microbiota is closely related to the prognosis of glioma. However, there is currently no corresponding bibliometric analysis to systematically review, analyze, and visualize prior research.

**Materials & methods:**

This study focused on 127 publications obtained from the Web of Science Core Collection and conducted correlation analyses of authors, journals, institutions, countries, keywords, and citations using VOSviewer, CiteSpace, and the R package.

**Results:**

The results indicate that China and the United States are the leading countries conducting research in this field. The Anderson Cancer Center at the University of Texas in the United States and Central South University in China are the primary research institutions.

**Discussion:**

Recent studies suggest that the regulation of the brain-gut axis between gut microbiota and glioma remains a prominent research hotspot, while the anti-cancer mechanisms of the enteric nervous system and gut microbiota have emerged as significant topics in recent years. Furthermore, the receptor tyrosine kinase pathway is expected to provide extensive research opportunities in the future. This article systematically reviews the current research status and hotspots of gut microbiota in glioma for the first time, conducts a visual analysis, and explores research trends and future directions.

## 1 Introduction

Glioma is currently the most prevalent primary central nervous system tumor globally, and its prognosis remains exceedingly poor. Among them, glioblastoma multiforme (GBM) has the poorest prognosis and the highest mortality rate. Existing treatment modalities, including surgery, chemotherapy, and radiotherapy, exhibit limited therapeutic efficacy against glioma. At best, these interventions can extend the survival of patients by only 15 months, and the recurrence rate is notably high (1). Due to the intricate structure of the brain and its physiological functions, the treatment of glioma has consistently posed a significant medical challenge (2). The World Health Organization classifies gliomas into four grades. Among these, glioblastomas (WHO grade IV) exhibit the highest degree of malignancy, the lowest survival rate, and are more prevalent in adults (3).

A substantial body of literature highlights the significant role of gut microbiota in the development of human cancers. The brain-gut axis, a recently discovered pathway connecting gut microbes and the brain, may influence the development of glioma by modulating the body’s immune response展 (4). Previous studies have documented the effects of gut microbiota on the body’s immune function and inflammation. The brain, immune cells, gut, and gut microbes are interconnected through the lymphatic system. Disruption of this system may contribute to the development and progression of glioma (5). In addition, several previous studies have found that the modulation of gut microbiota can affect the efficacy of immunotherapy (6). More researchers are increasingly focusing on the relationship between gut microbiota and glioma. Recent research has revealed a complex bidirectional communication between the brain and the gut microbiota, opening new avenues for regulating glioma development and treatment outcomes through manipulation of the gut microbiota. For example, alterations in the gut microbiota are closely linked to immune factors and inflammatory changes, both of which play critical roles in tumor initiation and progression. Therefore, enhancing glioma responsiveness to novel therapies by modulating the gut microbiota or tailoring treatment strategies based on the gut microbiota composition in glioma patients could significantly improve prognosis. However, the existing research is often unclear, and there is a lack of systematic analysis and visualization of bibliometric data. An in-depth bibliometric analysis of publications, countries, authors, journals, keywords, and other relevant factors is still necessary.

Bibliometrics involves the application of mathematical and statistical methods to systematically analyze the knowledge framework of a specific subject. This approach enables researchers to gain a clear understanding of current research hotspots and to evaluate the output capacity of authors, countries, and institutions. Consequently, bibliometrics can effectively illustrate the characteristics and research trends within a particular discipline. This study aims to systematically and comprehensively review the global research landscape of gut microbiota and glioma from 2005 to 2025, while also conducting the first bibliometric analysis in this area. Utilizing tools such as CiteSpace, VOSviewer, and the R package we can visualize scientific publications and map their interconnections. Through this systematic analysis, we aim to provide new treatment strategies and research directions for glioma, ultimately improving patient prognosis. Currently, research on the role of gut microbiota in the treatment of glioma has yielded preliminary results. However, there is no comprehensive, systematic review or knowledge network summarizing this field, nor is there an extensive analysis of research focuses and large-scale trends. This study conducts a bibliometric analysis to map research hotspots, collaboration patterns, and mechanistic themes linking gut microbiota and glioma, thereby guiding future research directions.

## 2 Method

### 2.1 Search strategy

The literature collected in this study was from the Web of Science Core Collection (wosCC). The search formula was TS=((“gut microbio “ OR “intestinal microbio “ OR “gut flora” OR “intestinal flora” OR “gut bacteri “ OR “intestinal bacteri “ OR “gut microbiota” OR “gut microbiome” OR “microbiota-gut-brain” OR “microbiome-gut-brain” OR “gut-brain axis” OR “brain-gut axis” OR “enteric nervous system” OR “gastrointestinal microbio “ OR “fecal microbio “ OR “gut dysbios “ OR “intestinal dysbios “) AND (glioma OR glioblastoma OR “brain tumor “ OR “brain tumour “ OR “brain neoplasm “ OR astrocytoma OR oligodendroglioma OR “neuroepithelial tumor “ OR “neuroepithelial tumour “ OR “GBM” OR “grade IV glioma” OR “high-grade glioma” OR “low-grade glioma” OR “diffuse intrinsic pontine glioma” OR “DIPG” OR “IDH-mutant” OR “IDH-wildtype”)), the article types were set to “article” and “review article”, the language was limited to “English”, and the search date was from January 1, 2005 to April 11, 2025. The detailed search process is shown in **Supplementary Material 1**.

### 2.2 Software for bibliometric analysis

In this study, we utilized Microsoft Office Excel 2003 to analyze the annual publication volume. VOSviewer1.6.2 and CiteSpace 6.2 R4 were employed to create visual maps based on the number of countries, institutions, journals, authors’ publications, and the frequency of keywords. Additionally, we analyzed co-cited references and highly cited references. The specific parameter settings and result interpretations in CiteSpace have been detailed in a previously published article (7). All articles published between January 1, 2005, and April 11, 2025, were selected for this study. The analysis was conducted using multiple key nodes, including country, institution, author, journal, keywords, and references. Thematic evolution was analyzed and visualized using the R package. Both VOSviewer and CiteSpace are powerful visualization analysis tools that help researchers uncover hidden knowledge structures, research hotspots, evolutionary trends, and academic relationships within vast amounts of literature data. These insights are difficult to achieve through traditional literature review and simple statistical methods. Moreover, by leveraging their robust data processing, network analysis, and visualization capabilities, these tools transform extensive literature collections into clear maps that reveal the structure and dynamics of scientific knowledge, significantly enhancing researchers’ insights and research efficiency.

## 3 Result

### 3.1 Analysis of annual published articles volume

**Figure 1:**
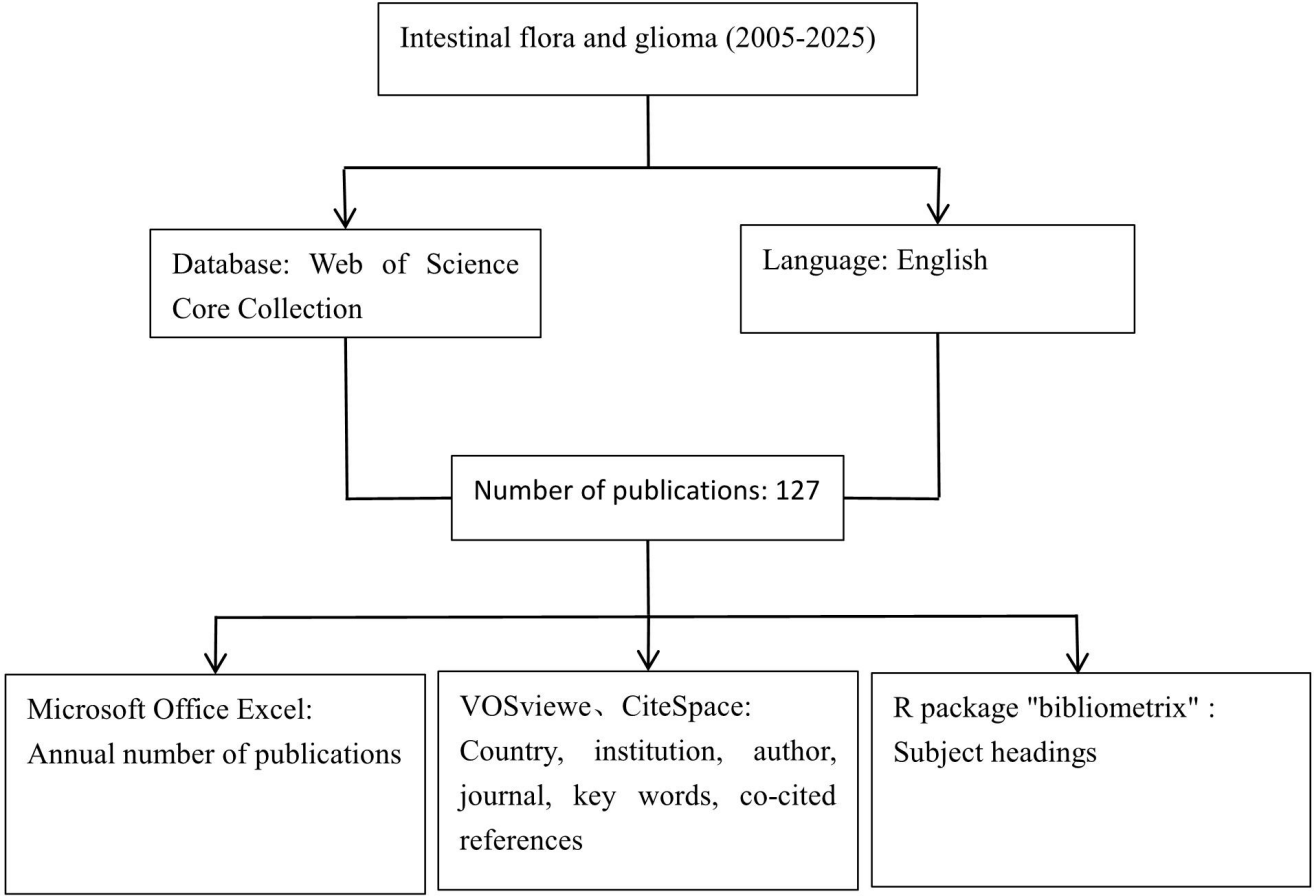
Research flowchart.

**Figure 2:**
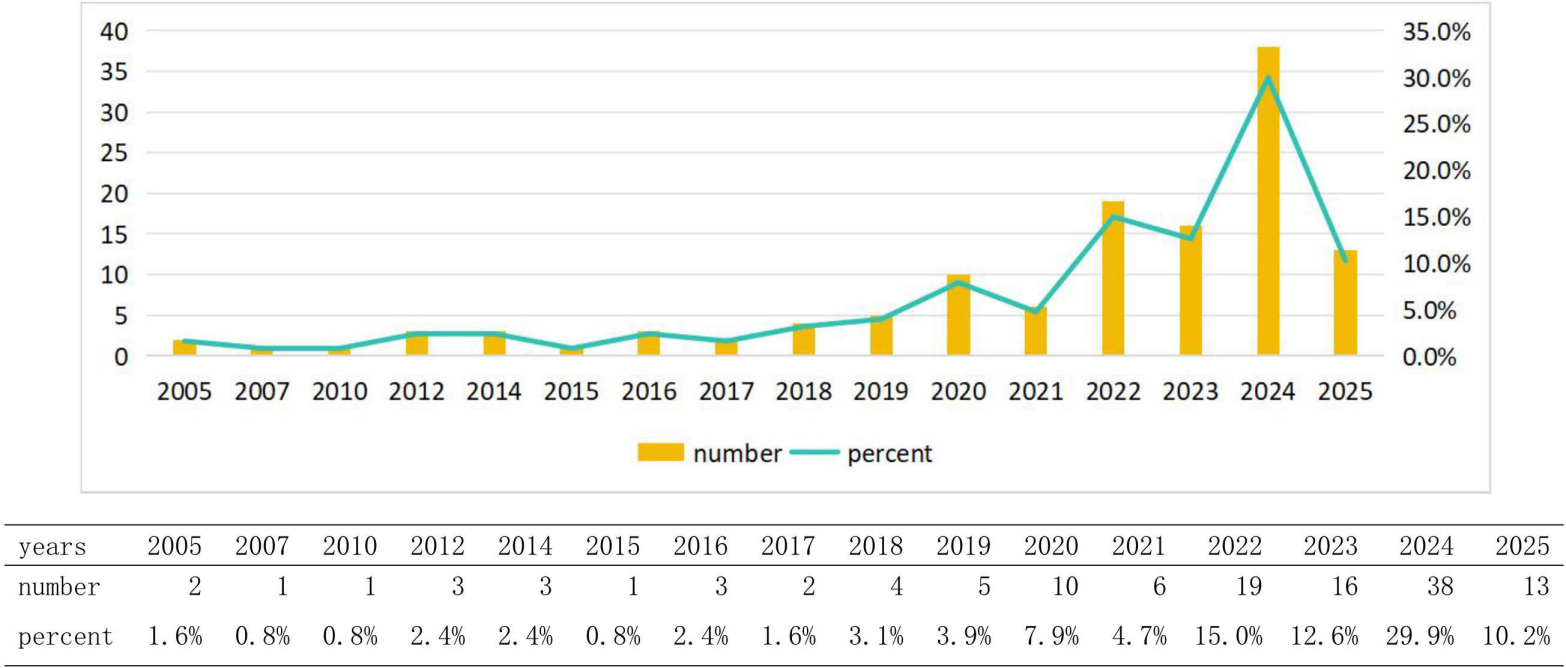
Chart of annual publications.

This study included a total of 127 valid studies, among which 43 were reviews and 84 were articles. The research process is illustrated in **Figure 1**, while the annual number of publications is depicted in **Figure 2**. Our findings indicate that research on gut microbiota and glioma from 2005 to 2019 was still in its early stages, with an annual publication volume of fewer than five articles. Since 2020, research on gut microbiota and brain glioma has shown a steady upward trend, particularly in 2024, which marked a period of explosive growth, reaching the highest publication volume recorded in any year. At the beginning of 2025, this discipline will continue to attract significant attention from researchers. This growth trend indicates that research on gut microbiota and brain glioma has been steadily progressing since 2020, experiencing a significant surge in interest within this field. Consequently, it is likely that more researchers will invest in this area of study.

### 3.2 Country analyses

**Figure 3:**
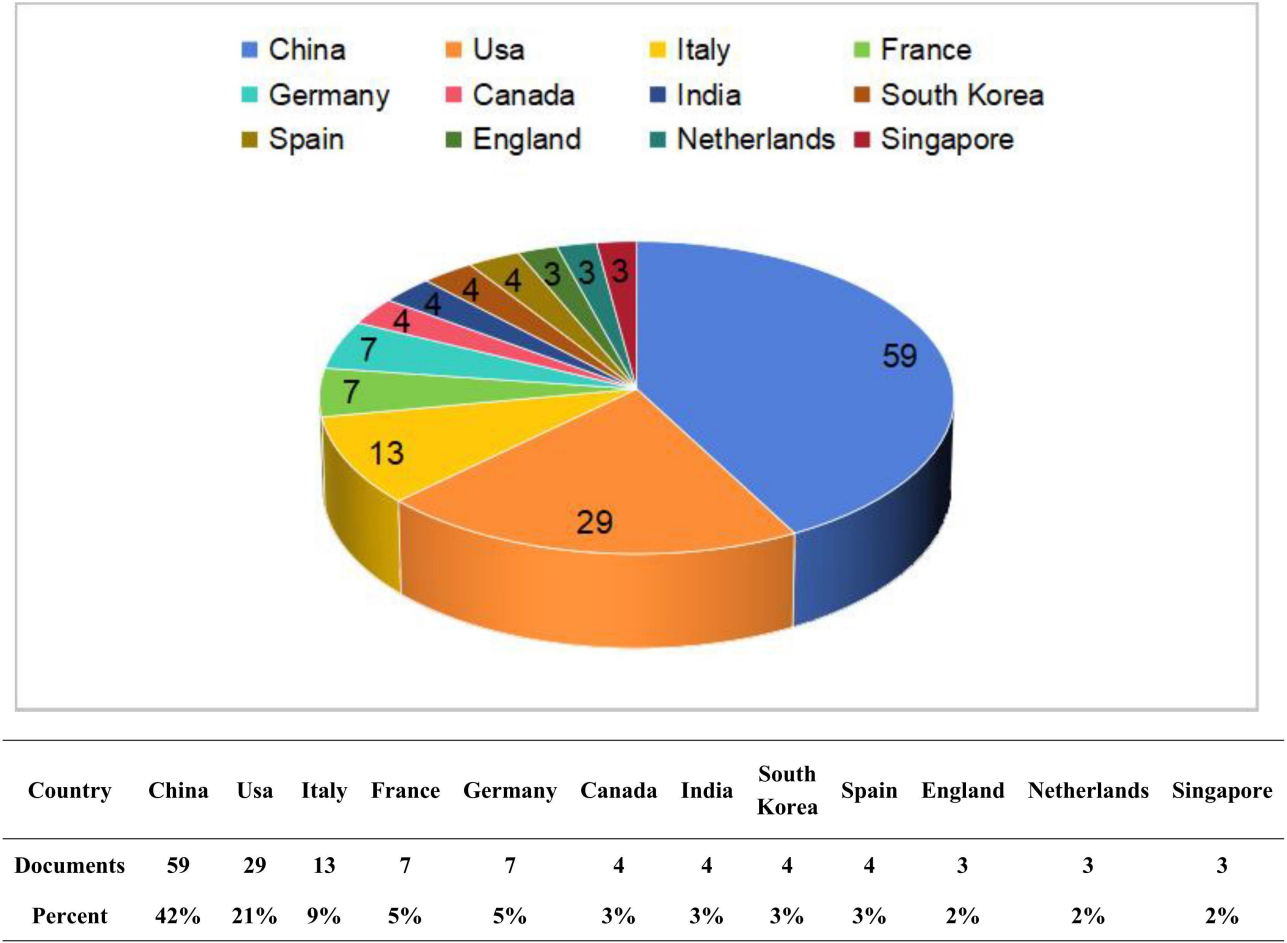
Chart of national publications.

**Figure 4:**
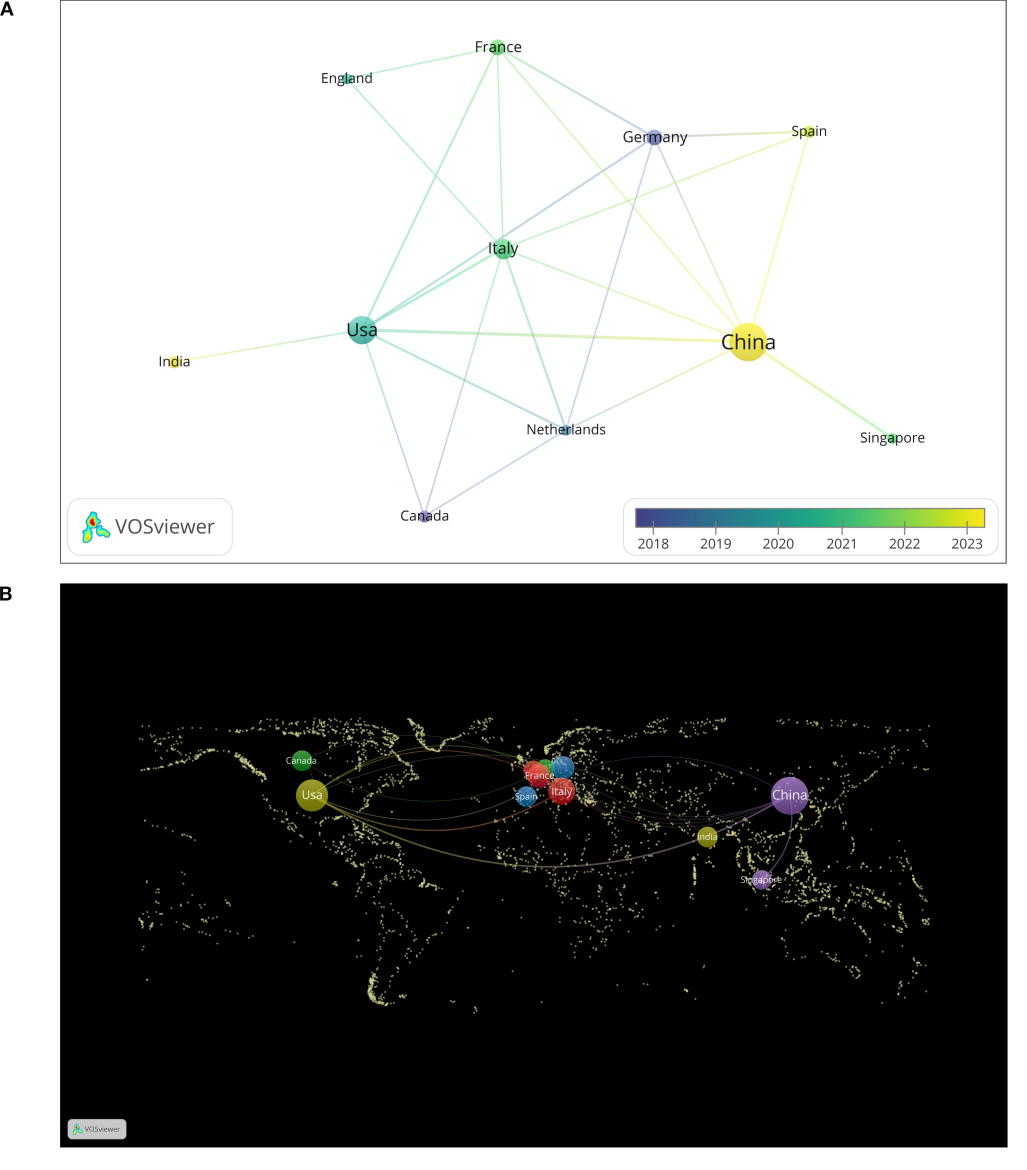
Chart of national publications.

Through the analysis of publications by country, we found that these publications originated from 41 different countries. **Figure 3** illustrates the top 12 countries with the highest number of publications. Among these countries, China (59 articles) and the United States (29 articles) produced the most significant body of literature, together accounting for more than half of the total publications. These two countries represent the leading authorities in this field and possess relatively mature research systems. Following them are Italy (13 articles), France (7 articles), and Germany (7 articles). We constructed a national cooperation network (**Figure 4A**) and a world map (**Figure 4B**) based on a minimum publication threshold of 3. China, the United States, Japan, Spain, the Netherlands, Italy, France, and Germany exhibit close collaborative ties. Additionally, the United States maintains strong cooperation with Canada, India, Italy, and France. Analyzing the cooperation network of the top 12 countries by publication volume reveals that India has collaborative relationships solely with the United States, while Singapore’s cooperation is limited to China. This suggests that extensive international collaboration has not yet been fully realized, likely due to geographical and linguistic barriers. In addition, countries such as the United Kingdom, the Netherlands, and Singapore have produced only a limited number of publications. This may be attributed to their lack of long-term engagement in such studies and the relative immaturity of their research processes. Therefore, countries with well-established research systems should engage in more extensive and in-depth academic exchanges with nations that are in the early stages of developing this field. Simultaneously, we recommend that additional countries participate in this research to further advance the development of this discipline.

### 3.3 Analysis of institution

**Figure 5:**
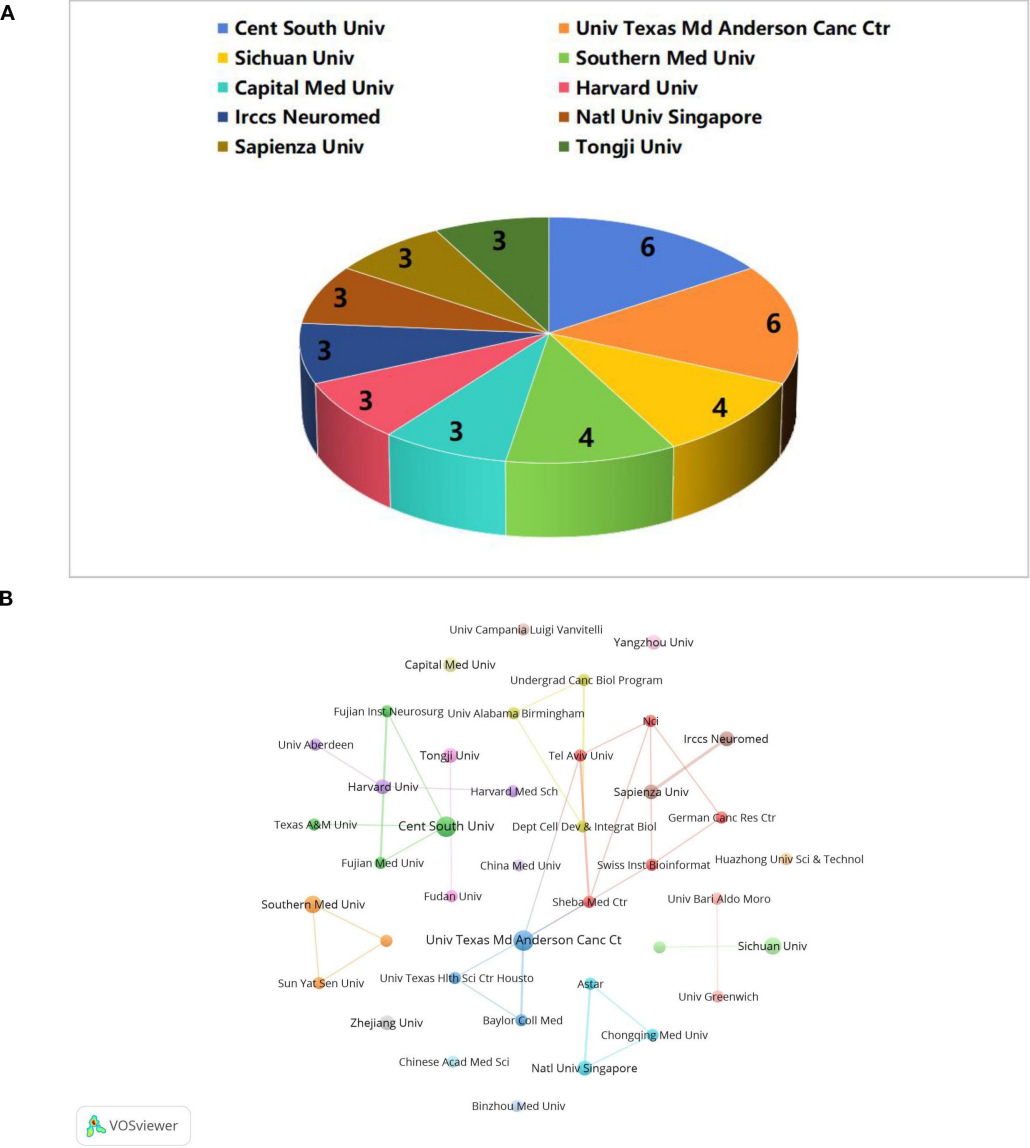
The number of publications by institutions **(A)** and cooperation with institutions **(B)**.

The literature for this study was collected from 322 institutions. Among the top 10 institutions with the highest number of publications (**Figure 5A**), the University of Texas MD Anderson Cancer Center in the United States and Central South University in China each contributed 6 publications. This was followed by Sichuan University in China and Southern Medical University, both of which published 4 articles. Thus, it is evident that China and the United States are the primary contributors to research in the field of glioma and gut microbiota. We constructed an inter-agency cooperation network diagram, setting the minimum number of publications at 2 (**Figure 5B**). In this diagram, the size of the nodes represents the number of publications, while the lines of varying colors indicate different cooperative groups. The University of Texas M.D. Anderson Cancer Center has established strong collaborations with Tel Aviv University, Sheba Medical Center, and other institutions. Central South University has formed partnerships with the Fujian Neurosurgical Institute, Fujian Medical University, Texas A&M University, among others. Southern Medical University has also developed a close relationship with Sun Yat-sen University. However, there has been limited collaboration among these various groups, likely due to insufficient academic exchanges stemming from geographical constraints. Furthermore, although Zhejiang University, Capital Medical University, and China Medical University have published numerous research papers, they have not established strong connections with other institutions. This lack of collaboration may be attributed to inadequate communication and cooperation in the early stages of research at each institution. Therefore, for future research endeavors, it is essential to enhance cooperation and knowledge sharing between domestic and international institutions to promote the advancement of this discipline.

### 3.4 Analysis of authors and co-cited authors

**Figure 6:**
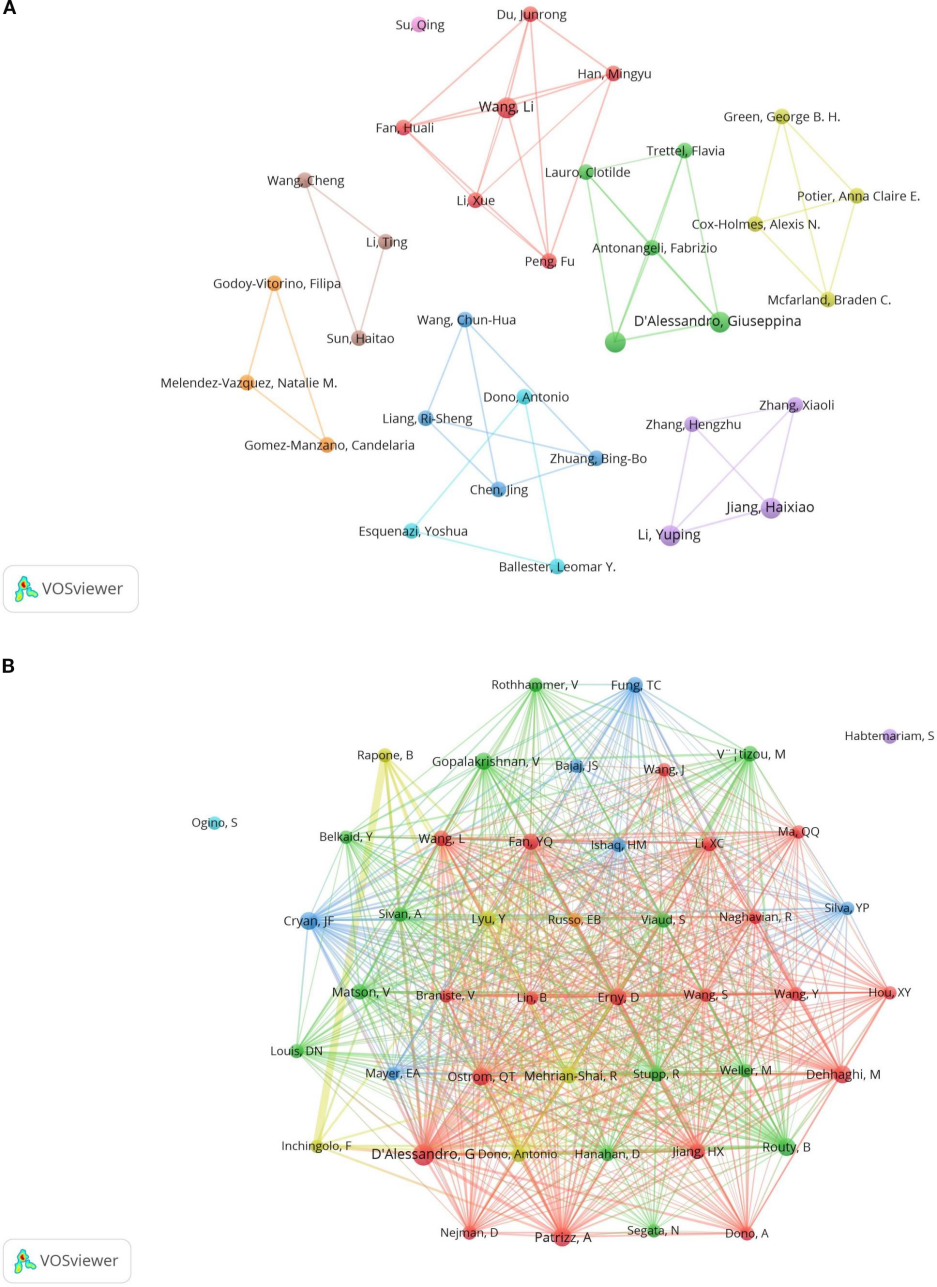
Network diagram of authors **(A)** and co-cited authors **(B)**.

A total of 891 authors participated in the study, with five of the top ten authors each publishing three articles (see **Table 1**). These authors are D’Alessandro Giuseppina, Jiang Haixiao, Li Yuping, Limatola Cristina, and Wang Li. We applied a filter based on a minimum publication volume of 2 and created a network illustrating the authors’ publication volumes (see **Figure 6A**). The five authors with the highest publication counts are represented by the largest nodes in the graph. The different colors in **Figure 6A** denote various collaborative groups. D’Alessandro Giuseppina, Antonangeli Fabrizio, and Lauro Clotilde exhibit close collaboration. Jiang Haixiao has closely worked with Li Yuping, Zhang Xiaoli, and Zhang Hengzhu. Wang Li has established a cooperative relationship with Li Xue and Han Mingyu. However, we observed a lack of communication between the different collaborating groups. Additionally, although Su Qing has published related papers, he has not collaborated with other authors. This lack of collaboration is detrimental to the advancement of the discipline, highlighting the need for enhanced academic exchanges among various authors and collaborative groups.

**Table 1:** Top 10 authors and co-cited authors of studies on glioma and gut microbiota.

Author	Documents	Co-cited authors	Citations
D’Alessandro, Giuseppina	3	D’Alessandro, G	46
Jiang, Haixiao	3	Patrizz, A	28
Li, Yuping	3	Cryan, Jf	26
Limatola, Cristina	3	Ostrom, Qt	25
Wang, Li	3	Routy, B	25
Antonangeli, Fabrizio	2	Dehhaghi, M	24
Ballester, Leomar Y.	2	Erny, D	21
Chen, Jing	2	Gopalakrishnan, V	21
Cox-Holmes, Alexis N.	2	Fan, Yq	20
Dono, Antonio	2	Jiang, Hx	18

In the co-cited author analysis, we found that the articles published by D’Alessandro, G were cited most frequently, with a total of 46 citations. This was followed by Patrizz, A with 28 citations and Cryan, JF with 26 citations. Their research forms the foundation of the discipline. The network was filtered and plotted with a minimum co-citation threshold of 10 (see **Figure 6B**), revealing close collaboration among various co-cited authors. There is a close co-citation relationship between D’Alessandro, G., Nejman, D., Qstrom, Q.T., Patrizz, A., Wang, L., et al.

### 3.5 Journal analysis

**Figure 7:**
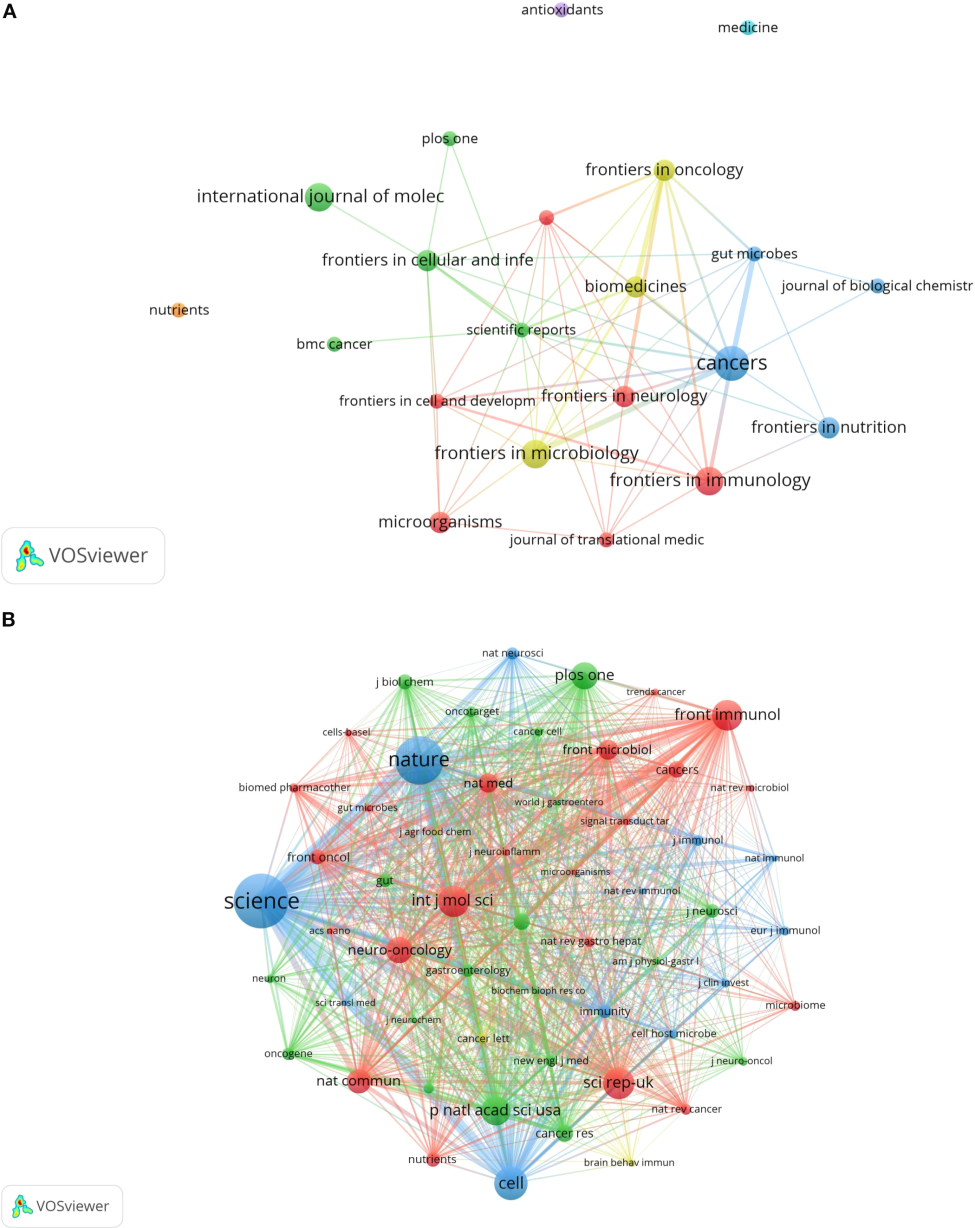
Network diagram of journals **(A)** and co-cited journals **(B)**.

**Figure 8:**
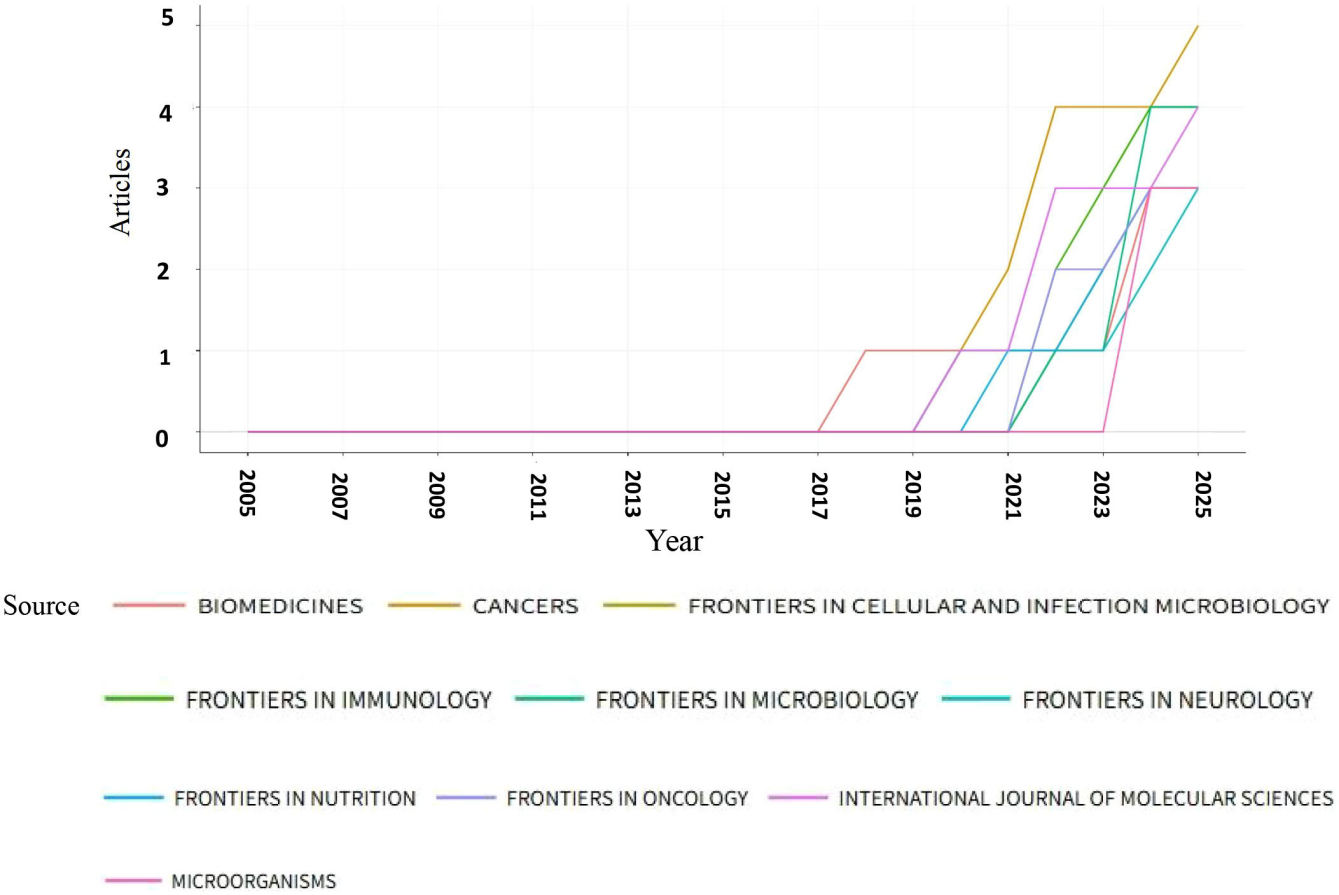
Annual publication volume of the top 10 journals in terms of publication volume.

From 2005 to 2025, a total of 91 journals published articles on the relationship between glioma and gut microbiota. The journal had the highest number of publications, with a total of 5 articles. This was followed by (4 articles), (4 articles), and the Journal of Molecular Sciences (4 articles). Based on the impact factor for 2024, the journal with the highest impact factor among the top 10 journals by publication volume is (IF 5.7), followed closely by the Journal of Molecular Sciences (IF 5.7). We established a minimum publication volume of 2 as a threshold to construct a journal network diagram (**Figure 7A**). The results indicated that the Cancers exhibited strong citation relationships with Frontiers in Immunology, Frontiers in Nutrition, Scientific Reports, Biomedicines, and others. The top 10 journals and co-cited journals relevant to glioma and gut microbiota research are presented in **Table 2**. Additionally, journals such as Medicine and Nutrition have not established citation relationships with other journals, despite having published relevant literature. A further analysis of the annual publication volume of these journals is illustrated in **Figure 8**, which shows that the top 10 journals began publishing research in this field in 2017. Notably, Biomedicines was the first journal to publish studies in this area, while Cancers had the highest annual publication volume after 2019.

**Table 2:** Top 10 journals and co-cited journals for glioma and gut microbiota research.

Source	Documents	IF	Co-citation source	Citations	IF
Cancers	5	4.5	Science	270	44.8
Frontiers In Immunology	4	5.7	Nature	238	50.5
Frontiers In Microbiology	4	4.0	Cell	164	45.6
International Journal Of Molecular Sciences	4	4.9	INTERNATIONAL JOURNAL OF MOLECULAR SCIENCES	155	4.9
Biomedicines	3	3.9	Scientific Reports	155	3.8
Frontiers In Cellular And Infection Microbiology	3	4.6	Frontiers in Immunology	151	5.7
Frontiers In Neurology	3	2.7	PROCEEDINGS OF THE NATIONAL ACADEMY OF SCIENCES OF THE UNITED STATES OF AMERICA	145	9.4
Frontiers In Nutrition	3	4.0	PLoS One	135	2.9
Frontiers In Oncology	3	3.5	Neuro-Oncology	129	16.4
Microorganisms	3	4.1	Nat Commun	119	14.7

The results of the co-citation analysis indicated that the most frequently cited journal was Science (270 citations), followed by Nature (238 citations). Among the top ten cited journals, three had an impact factor exceeding 40: Nature (IF 50.5), Cell (IF 45.6), and Science (IF 44.8). These journals exert significant influence within the scientific community and represent the forefront of research in this discipline. A minimum co-citation frequency of 30 was established as the threshold for constructing the journal co-citation network (**Figure 7B**). The analysis revealed a very strong co-citation relationship between Nature and Science, as well as Cell, Immunity, and others.

### 3.6 Co-cited references and citation outbreaks

**Figure 9:**
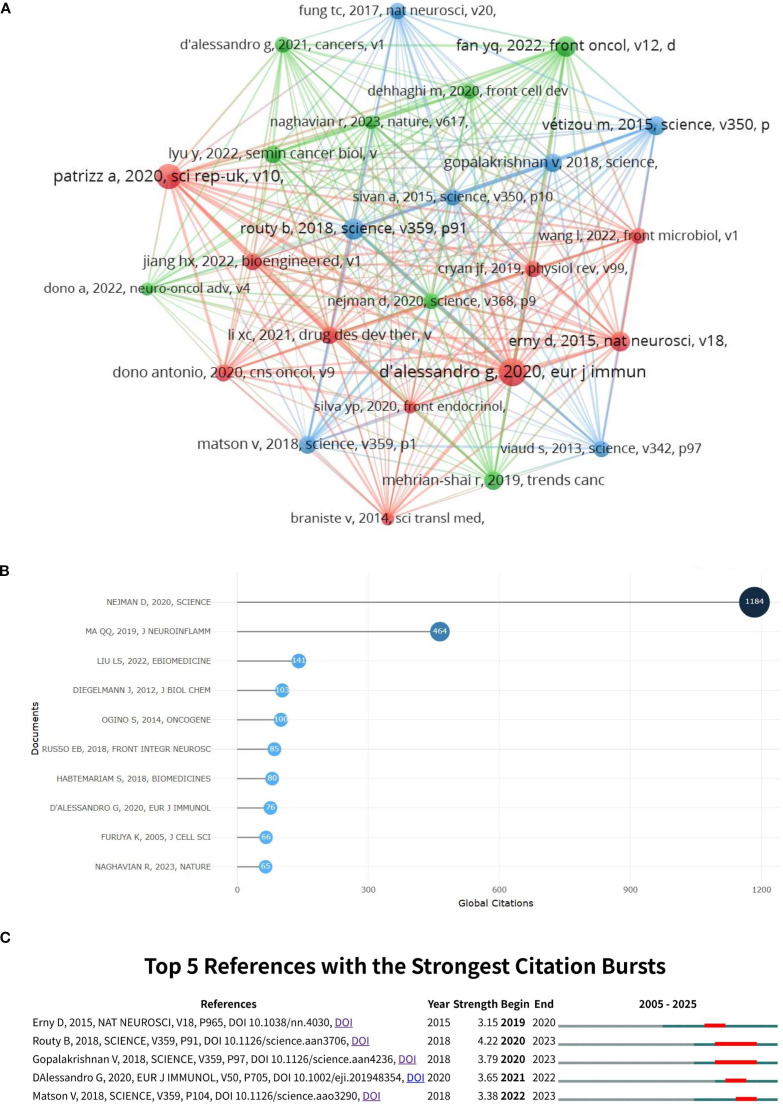
Visualization of co-cited references for glioma and gut microbiota **(A)** the top 10 co-cited references **(B)** and the top 5 citation bursts **(C)**.

A total of 9,387 references related to glioma and gut microbiota were found during the period from 2005 to 2025. The co-cited reference network was constructed using VoSviewer with a minimum co-citation count of 10 (**Figure 9A**). The top 10 co-cited references were analyzed using the R package bibliometrix (**Figure 9B**), and the top 5 references with citation bursts were analyzed using CiteSpace (**Figure 9C**). The titles, publication years and main contents of the top three cited articles in **Figure 9B** are shown in **Supplementary Material 3**. As shown in **Figure 9B**, the top 10 cited references all had more than 60 citations. In **Figure 9A**, “Patrizia, 2020, Sci Rep-UK, v10” had a strong co-citation relationship with “Jiang HX, 2022, Bioengineered, v1”, “Dono Antonio, 2020, CNS Oncol, v9”, etc. **Figure 9C** visualized the top 5 references with citation bursts, with the highest burst intensity of 4.22, representing the highest citation popularity during this period. **Table 3** presents the detailed information of the top 5 references with citation bursts.

**Table 3:** The references of the top 5 citation bursts.

Publications	Strength	Author	Journal	IF
Gut microbiome influences efficacy of PD-1-based immunotherapy against epithelial tumors	4.22	Bertrand Routy	Science	44.8
Gut microbiome modulates response to anti-PD-1 immunotherapy in melanoma patients	3.79	V Gopalakrishnan	Science	44.8
Gut microbiota alterations affect glioma growth and innate immune cells involved in tumor immunosurveillance in mice	3.65	Giuseppina D’Alessandro	European Journal of Immunology	4.5
The commensal microbiome is associated with anti-PD-1 efficacy in metastatic melanoma patients	3.38	Vyara Matson	Science	44.8
Host microbiota constantly control maturation and function of microglia in the CNS	3.15	Daniel Erny	Nature Neuroscience	21.2

### 3.7 Keyword analysis

**Figure 10:**
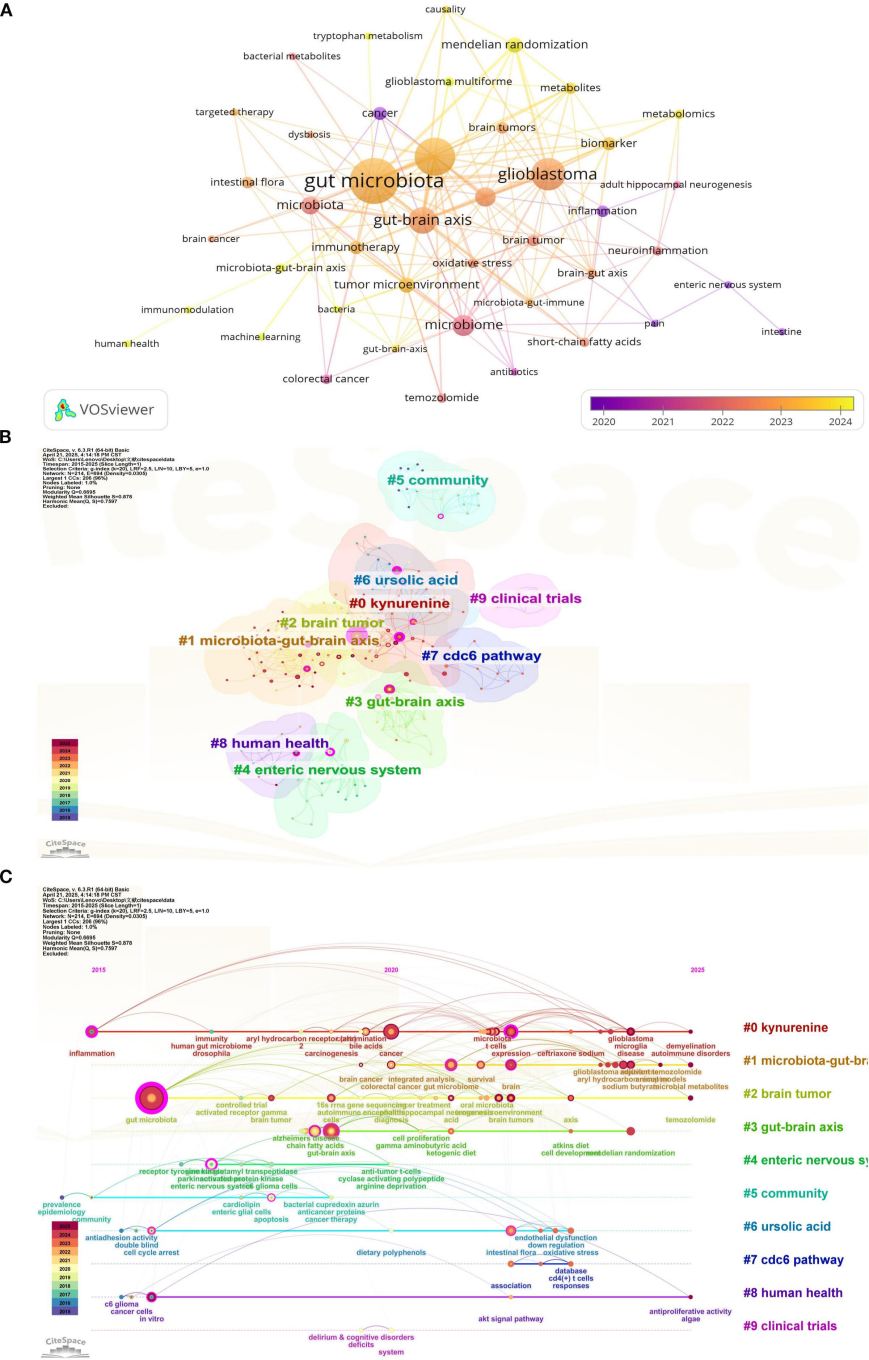
Keyword network diagram constructed by VOSviewer **(A)**, keyword cluster analysis visualized by CiteSpace **(B)** and keyword timeline view **(C)**.

Keyword analysis allows us to quickly understand the current main research directions and research hotspots. **Table 4** presents the top 20 keywords. Among these keywords, gut microbiota, glioma, glioblastoma, gut-brain axis, microbiome appeared more than 10 times. gut microbiome, microbiota, mendelian randomization, tumor microenvironment, biomarker frequency ≥ 5 times. These keywords represent the primary directions and research hotspots related to glioma and gut microbiota. We established a minimum frequency threshold of 2 to construct a keyword network diagram (**Figure 10A**). Larger nodes indicate more frequent occurrences, while thicker lines represent stronger correlations. This visualization intuitively displays the keywords associated with the topic, facilitating further analysis of the subject matter for researchers.

**Table 4:** Top 20 keywords in the study of glioma and gut microbiota.

Rank	Keyword	Occurrences	Rank	Keyword	Occurrences
1	gut microbiota	35	11	cancer	5
2	glioma	26	12	immunotherapy	5
3	glioblastoma	20	13	brain tumor	4
4	gut-brain axis	15	14	brain tumors	4
5	microbiome	10	15	inflammation	4
6	gut microbiome	9	16	intestinal flora	4
7	microbiota	8	17	metabolites	4
8	mendelian randomization	6	18	brain-gut axis	3
9	tumor microenvironment	6	19	colorectal cancer	3
10	biomarker	5	20	glioblastoma multiforme	3

Through the analysis of the frequency and time of keywords, we find out the most meaningful keywords that can reflect the research hotspots, and draw the keyword cluster diagram and time axis diagram. Keyword clustering (**Figure 10B**) and timeline (**Figure 10C**) plots can reveal the research basis of this discipline. After the analysis through citespace, the clusters we obtained were mainly #0 kynurenine, #1 microbiota-gut-brain axis, #2 brain tumor, #3 gut-brain axis, #4 enteric nervous system, and #5 community, #6 ursolic acid, #7 cdc6 pathway, #8 human health, #9 clinical trials. The timeline plot illustrates the evolution of research hotspots over time, with items positioned closer to the right indicating more recent developments. The clustering labels on the timeline correspond to our keyword clustering, suggesting that current research hotspots primarily focus on analyzing the potential mechanisms by which gut microbiota regulate glioma prognosis through the gut-brain axis, immune regulation, and other pathways.

### 3.8 Thematic maps

**Figure 11:**
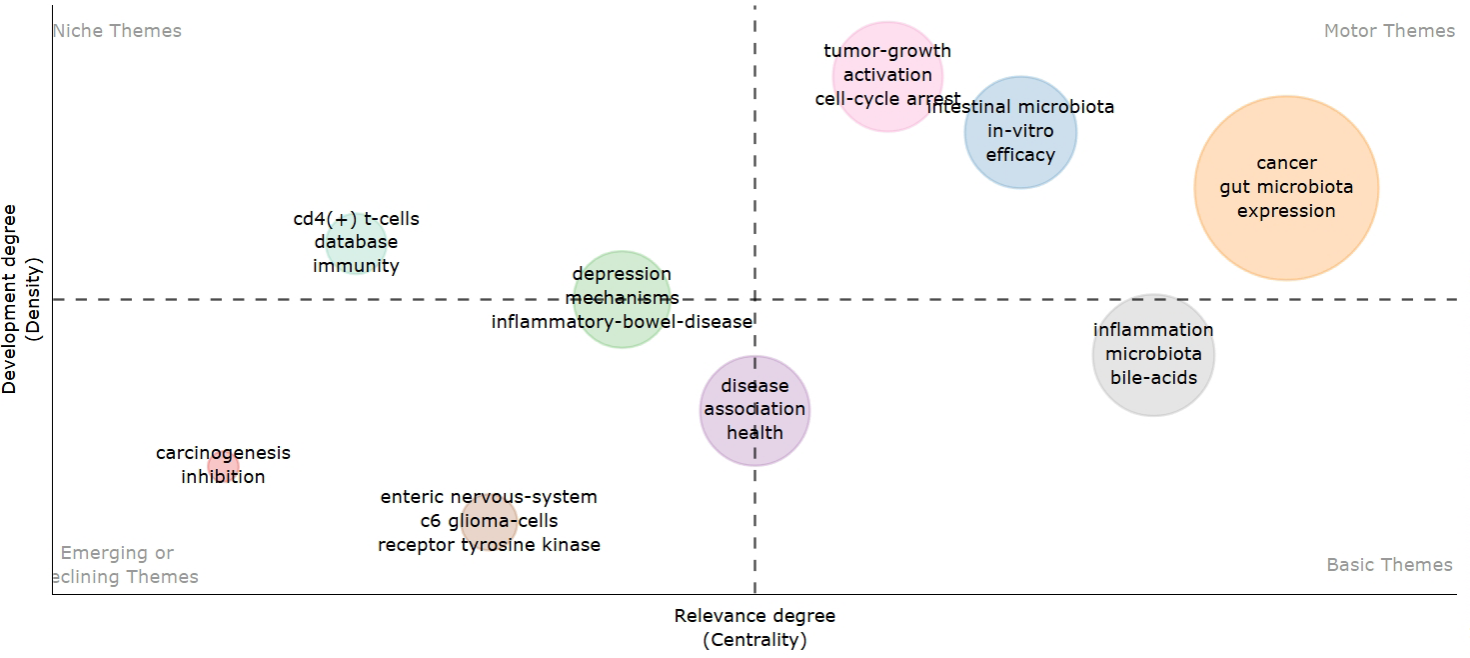
Thematic map based on keywords.

Thematic maps are designed to illustrate the internal relationships and the strength of connections among various topics within the discipline. Centrality, represented on the horizontal axis, indicates the correlation between topics; a higher centrality signifies greater importance of a topic within the field. Density, depicted on the vertical axis, reflects the strength of links between keywords associated with a given topic, with higher density suggesting more advanced research. As illustrated in **Figure 11**, topics such as tumor growth activation, cell cycle arrest, the *in vitro* efficacy of intestinal microbes, and the expression of intestinal microbes in cancer have been relatively well developed and hold significant relevance in this discipline. Emerging topics, including carcinogenesis inhibition, the enteric nervous system, and receptor tyrosine kinase, present promising research opportunities for the future and warrant our attention.

## 4 Discussion

### 4.1 General information

This study included a total of 127 valid studies, among which 43 were reviews and 84 were articles. Articles and reviews are the most important types of documents analyzed in bibliometrics. They are the most common and standardized document types in international mainstream journals, featuring consistent formats and publication procedures. These documents represent the core knowledge and integration within a discipline. Articles serve as the cornerstone of scientific knowledge growth, presenting the latest research findings, methods, and data. They directly reflect the frontiers and active research areas of the field. Reviews, on the other hand, provide systematic summaries, critical evaluations, and syntheses of research progress in a specific area over a given period. They reveal research trends, knowledge structures, hotspots, and future directions, making them essential for understanding the maturity and evolution of a discipline. While articles deliver the most recent research findings, reviews organize and integrate these results, offering guidance for future research trends and directions. The combination of both types of documents drives the advancement of the discipline.

Since 2020, research on glioma and gut microbiota has garnered significant attention from scholars, with a steady increase in the annual number of publications, particularly in 2024. This trend indicates that over the past five years, the foundational theories in related disciplines have matured, leading to a substantial enhancement in research productivity within this field, aided by advancements in scientific methodologies. Over the last two decades, there has been a clearer understanding of the intrinsic relationship between gut microbiota and glioma. Variations in intestinal microecology among individuals can influence cancer phenotypes (8). Various exchanges of information between gut microbes and the brain occur through the gut-brain axis (9), and alterations in gut microbiota are closely associated with central nervous system diseases (10). New research has found that gut microbiota is associated with the pathogenesis of glioma. Additionally, alterations in gut microbes are often observed in patients with glioma, potentially resulting from bidirectional signaling within the gut-brain axis (9). Therefore, based on this unique physiological phenomenon, researchers are striving to enhance the therapeutic effects of glioma through the modulation of intestinal microbiota. To this end, scientists worldwide have initiated comprehensive analyses. China and the United States are the primary countries conducting this research, and there is significant collaboration between them. China has published 59 papers, while the United States has published 29. China’s publication volume is significantly higher than that of other countries, with the United States ranking second. This data indicates that research in this field by China and the United States has reached a relatively mature stage. These studies will provide the latest theoretical frameworks for other countries to conduct similar research. In the future, China and the United States should lead international collaborations to promote the further development of this discipline. Various collaborative groups have been established in both countries; however, some nations have not participated in this research. Therefore, it is essential to establish more extensive and profound academic exchanges among countries worldwide to advance communication in this discipline. From the perspective of institutional analysis, the primary research institutions are the University of Texas M.D. Anderson Cancer Center in the United States and Central South University in China; however, there is currently no cooperative relationship between them. Furthermore, institutions globally are relatively fragmented and have not established comprehensive exchanges and collaborations, which hinders progress in this field. Institutional cooperation serves as a crucial driving force for the advancement of the discipline. Research institutions should actively seek comprehensive partnerships and enhance international exchanges to improve their competitiveness. For example, Jennifer Wargo’s team at MD Anderson in the United States has made a breakthrough in collaboration with the Department of Neurooncology in China. They revealed the mechanism by which intestinal flora regulates immune suppression in the glioma microenvironment through tryptophan metabolism (kynurenine pathway) (31), and also found that tumor-promoting bacteria such as Fusobacterium nucleatum may cross the blood-brain barrier (BBB) to affect brain tumors (21). The GLIOTRAIN consortium (comprising eight European countries and Australia) has validated the predictive value of microbiota markers (e.g., butyrate-producing bacteria abundance) for temozolomide chemotherapy response, which has advanced microbial intervention strategies into phase II clinical trials (e.g., probiotic-assisted radiotherapy). These examples of international cooperation highlight the significant value of transnational collaboration. However, due to technical barriers and differing scientific perspectives among countries, communication and cooperation remain limited. To address these challenges, developed countries can lead funding efforts and influence journal policies, while developing countries can contribute extensive patient resources and receive co-authorship rights for samples provided. This collaborative approach between developed and developing countries can jointly promote the advancement of this discipline.

### 4.2 Author analysis

From the author analysis, it is evident that D’Alessandro Giuseppina, Jiang Haixiao, Li Yuping, Limatola Cristina, and Wang Li have published the most articles. D’Alessandro Giuseppina and Limatola Cristina authored a paper suggesting a bidirectional interaction between the gut microbiome and glioma. The gut microbiota influences the function of the central nervous system by producing and regulating neurotransmitters such as dopamine, serotonin, glutamate, and gama-aminobutyric acid (GABA), and may play a role in the onset and progression of glioma. The authors proposed that gut microbiota may regulate the proliferation, differentiation, and immune microenvironment of glioma cells through neural signal transmission mediated by the gut-brain axis (11). In the future, it will be necessary to further explore the potential pathways through which specific microbial communities regulate neurotransmitters. Additionally, there is a need to modulate intestinal microbiota through dietary interventions, probiotics, or fecal microbiota transplantation, as well as to develop adjuvant therapies for glioma that target neurotransmitter pathways. Furthermore, the specific roles of neurotransmitters in the tumor microenvironment and their potential for clinical translation must be elucidated. A study conducted by Jiang Haixiao and Li Yuping investigated the potential role of gut microbiota in glioma using human samples. Studies have shown that the gut of patients with malignant brain tumors is rich in carcinogenic bacteria, such as Fossoriella and Akkermansia. However, the abundance of beneficial short-chain fatty acid (SCFA) -producing bacteria, including Laknosella, Agarobacter and Bifidobacterium, decreased. The study also developed a combination of microbial markers based on specific bacterial genera, which had significant diagnostic efficacy. In addition, the study revealed potential pathways by which gut microbes contribute to brain tumor formation through metabolism, such as glutamate metabolism (12). Regulation of gut microbiota, including the supplementation of probiotics and fecal microbiota transplantation, may emerge as a novel strategy for the adjuvant treatment of glioma. Nejman et al. demonstrated the presence of microorganisms, such as Bifidobacterium, in gliomas through their research (13). Wang Li investigated the inhibitory effects of Bifidobacterium on glioma and its underlying mechanisms. Multi-omics analysis revealed that Bifidobacterium exerts a comprehensive effect in inhibiting tumor growth by regulating both tumor and intestinal microbiota, inhibiting the MEK/ERK signaling pathway, and altering serum metabolites, including organic nitrogen compounds. Wang Li found that Bifidobacterium significantly reduced tumor volume and prolonged the median survival time of mice (14). Wang Li’s study provides a robust experimental foundation for the development of probiotic-based therapeutic strategies for glioma. Based on the findings above, we propose that glioma treatment interventions can be approached through the following strategies. It has been demonstrated that bacteria producing short-chain fatty acids can regulate the expansion of regulatory T cells (Tregs) in animal models, suggesting that fiber supplementation or interventions targeting short-chain fatty acid pathways may have therapeutic potential. The ketogenic diet has been shown to enhance the efficacy of certain targeted therapies, such as PI3K/mTOR and EGFR inhibitors, in glioma models, indicating that mimicking the ketogenic diet could be beneficial as an adjuvant treatment for glioma. Additionally, some probiotic strains may directly or indirectly (via metabolites) modulate immune system function, and increasing the proportion of beneficial bacteria may further support adjuvant glioma therapy. However, the use of diet, microbiota, and metabolic regulation to enhance targeted glioma treatments remains a promising yet exploratory area. Further clinical studies are necessary to validate these approaches.

### 4.3 Journal publication analysis

Analyzing journal publications assists researchers in identifying appropriate journals within their field for article submission. Peer review is a crucial step in the publication process. Biomedicines was the original journal to publish this study, while Cancers has been the journal with the largest annual publication volume since 2019. The most frequently cited journal is Science, which has received 270 citations and boasts an impact factor of 44.8. The impact factor reflects a journal’s influence within its discipline. Generally, journals with higher impact factors are cited more frequently. Data from Science indicates that articles published in this journal represent the most authoritative research findings in the field and hold significant reference and guiding value for subsequent studies. Cancers published the largest number of papers in this area, with five articles and an impact factor of 4.5. The number of publications reflects the journal’s popularity. The high volume of publications by Cancers suggests that the journal is large in scale, publishes papers rapidly, and facilitates the dissemination of research results in this field. Therefore, selecting a journal based on both its impact factor and publication volume is more conducive to the dissemination and advancement of research in this discipline.

### 4.4 Co-cited reference analysis

Co-cited references are those that are cited together by researchers. Analyzing co-cited references has enabled us to identify foundational research in this field. In this study, we utilized VOSviewer to examine the citation relationships among co-cited references. Using VOSviewer, we categorized the co-cited references into three distinct clusters. The three different color categories represent different research modalities and research models.

The red cluster is dominated by animal models.The red cluster emphasizes the interaction between glioma and gut microbiota. A study has found that long-term antibiotic treatment can promote glioma growth by altering the composition of gut microbiota and modulating the immune status of the brain (15). In addition, another study found that glioma growth resulted in an increase in gut microbiota belonging to the phylum Verrucomicrobia and the genus Akkermansia, which was inhibited by temozolomide treatment (16).

The green cluster was dominated by cell experiments and cell models were established by cell culture for study. Neurotransmitters such as dopamine, serotonin, and glutamate play a crucial role in the invasion and growth of gliomas. These neurotransmitters are produced through the activity of intestinal flora (17). The study of green cluster mainly analyzes the role of gut microbiota on glioma at the small molecule level. Studies have demonstrated that gut microbiota can influence neurotransmitters in the brain, including dopamine, norepinephrine, serotonin, GABA, acetylcholine, histamine, and tryptophan metabolites, which in turn affect the proliferation of glioma (11). One report indicated that serotonin stimulates the proliferation of glioma cells (18). A separate study demonstrated that GABA inhibits the growth of low-grade gliomas (19). In addition, a recent study found that bacterial peptides derived from microorganisms can activate tumor-infiltrating immune cells in glioblastoma, providing strong evidence for the development of glioma vaccines (20).

The blue cluster is dominated by human studies. Immunotherapy plays a crucial role in tumor treatment. The blue cluster emphasizes the potential application of gut microbiota in cancer immunotherapy. A study has demonstrated that the composition of the gut microbiota can modify the permeability of the blood-brain barrier (BBB) and influence the effectiveness of tumor immunotherapy within the tumor immune microenvironment (21). Therefore, gut microbiota-based immunotherapy represents a promising therapeutic approach for glioma.

In bibliometrics, highly cited meta-analyses and systematic reviews serve as core knowledge nodes and domain shapers, profoundly influencing the formation, identification, and thematic evolution of co-cited clusters. These highly cited review articles typically provide comprehensive summaries, integrations, and critical evaluations of previous research within a specific field. By synthesizing the best findings from numerous related studies, they become one of the most important sources of knowledge for subsequent research. Such influential meta-analyses and systematic reviews guide future investigations to focus on identified gaps or emerging questions, thereby shaping the evolutionary trajectory of the field. When mapping scientific knowledge, the publication of highly cited reviews often marks a critical juncture in the development of a research topic. The most co-cited reference is an article by Deborah Nejman published in Science in 2020 (DOI: 10.1126/science.aay9189). The study has been cited 1,184 times. In her study, Nejman conducted a systematic analysis of 1,526 samples from seven different human tumor types and discovered that each tumor type exhibited distinct combinations of microbes (13). The characterization of tumor-associated microorganisms is essential for understanding the influence of tumor bacteria on tumor markers. Additionally, analyzing these microorganisms aids in tumor identification. This research has provided a clear direction for future studies. Building on this foundation, subsequent researchers have further investigated the impact of gut microbial composition on tumors, revealing its significant role in tumor growth, proliferation, and immunotherapy. Currently, research in this field has emerged as a focal point in glioma treatment.

The second most frequently cited article was Qianquan Ma’s study published in the Journal of Neuroinflammation in 2019 (DOI: 10.1186/s12974-019-1434-3). The article has been cited 464 times. This study highlighted the bidirectional interaction between gut microbiota and the brain, providing a detailed discussion of the brain-gut axis. It established a close biological connection among the microbiota, immune signaling, and the central nervous system, demonstrating that microbial metabolites can modulate neural and immune activity in the brain (22). In addition, one study suggested that gut microbiota produce CK (a ginsenoside metabolite) after the consumption of ginseng, which may inhibit glioma proliferation and invasion by disrupting the downstream SDF-1 and CXCR4 signaling pathways (23). These studies offer a more precise research framework and foundation for future researchers to investigate the relationship between glioma and intestinal flora.

Citation bursts reflect the most frequently cited references over a specific period. Citation outbreak analysis enables us to quickly identify research hotspots and foundational studies within a given timeframe. Through this analysis, we have determined that intestinal microbiota-based immune regulation is a primary focus of current research. A study conducted by Giuseppina D’Alessandro suggests that dysbiosis of gut microbiota promotes glioma development by impairing natural killer (NK) cell function and inducing an immunosuppressive phenotype in microglia, thereby compromising tumor immune surveillance in the central nervous system (CNS) (15). However, the potential pathways by which intestinal flora regulate glioma immunity remain unclear, and further experiments are necessary to explore the impact of intestinal flora on immune activity.

In conclusion, through co-cited reference analysis, we have gained a comprehensive understanding of the potential pathways by which gut microbiota influence glioma, which can be summarized as follows. Human studies: (i) a decrease in beneficial gut SCFA-producing bacteria increases the risk of glioma; (ii) the composition of gut microbiota can alter blood-brain barrier (BBB) permeability and affect the efficacy of tumor immunotherapy within the tumor immune microenvironment. Cellular studies: (i) serotonin produced by gut microbiota stimulates glioma growth; (ii) γ-aminobutyric acid produced by gut microbiota significantly inhibits glioma growth. Animal studies: (i) long-term antibiotic administration promotes glioma growth by altering gut microbiota composition and modulating the brain’s immune status. The identification of these potential pathways provides valuable references for researchers and establishes a solid foundation for advancing this field.

### 4.5 Key words and hotspot analysis

Through keyword analysis, we can swiftly identify current research hotspots. After filtering out basic keywords such as “gut microbiota, glioma, glioblastoma”, we found that the gut-brain axis and mendelian randomization appeared most frequently. “gut-brain axis” occurred 15 times, and “mendelian randomization” occurred 6 times. High-frequency keywords reveal the core themes and research focus of the research field. Through the keyword frequency analysis, we know that “gut-brain axis” is the focus of the research on gut microbiota and glioma, and is also the most valuable direction in this field. Mendelian randomization analysis emerged as the predominant research method for studying gut microbiota and glioma. Utilizing keyword clustering and a timeline map, we organized and categorized the keywords, resulting in the re-clustering of all keywords into ten distinct modules. These modules are as follows: #0 Kynurenine, #1 Microbiota-Gut-Brain Axis, #2 Brain Tumor, #3 Gut-Brain Axis, #4 Enteric Nervous System, #5 Community, #6 Ursolic Acid, #7 CDC6 Pathway, #8 Human Health, and #9 Clinical Trials. The kynurenine pathway is the primary route of tryptophan metabolism, and any abnormalities in kynurenine metabolism can contribute to the progression of brain tumors (24). A study conducted by Mona Dehhaghi demonstrated that gut microbes deplete tryptophan, induce immune tolerance, and enhance tumor cell viability through the phosphorylation of eukaryotic initiation factor 2α (25). When tryptophan is depleted, the environmental non-dissuppressor 2 (GCN2) becomes overactive, disrupting fatty acid synthesis in CD4+ T cells. This disruption can lead to abnormal proliferation and function of effector T cells, which may facilitate tumor proliferation and growth. Conversely, intestinal microorganisms can convert tryptophan into kynurenine, leading to hyperactivation of the aryl hydrocarbon receptor (AhR). This hyperactivation, in turn, stimulates the activity of indoleamine 2,3-dioxygenase (IDO-1) through the release of IL-6 and IFN-γ. Excessive activation of IDO-1 results in further depletion of tryptophan. The kynurenine pathway may represent a potential mechanism by which gut microbes promote glioma growth. In addition, the biological regulation of the gut-brain axis has been proposed to influence the development and function of the central nervous system (26). Intestinal microbial activities can produce GABA and influence brain activity by modulating the immune response (27). This may represent another potential mechanism through which intestinal microbes affect glioma growth. Through the temporal analysis, we identified microbial metabolism, amino acid metabolism, and temozolomide as the primary research hotspots in this field in recent years, warranting further investigation. Thematic maps revealed that topics related to tumor growth activation, cell cycle arrest, the *in vitro* efficacy of intestinal microbes, and the expression of intestinal microbes in cancer have been relatively well developed within our discipline. Additionally, carcinogenesis inhibition, the enteric nervous system, and receptor tyrosine kinase are emerging topics in this area of study. Cancer is a multi-disciplinary and multi-stage developmental process. Recent studies have shown that intestinal microorganisms can influence the occurrence and progression of cancer through various potential pathways (28). Additionally, the impact of intestinal flora on glioma underscores the significance of the gut-brain axis in brain function (4). Previous studies have demonstrated that metabolites generated by intestinal microbial activity influence tumor progression primarily by altering the tumor microenvironment and modulating signaling pathways in various immune cells (29). A study demonstrated that bifidobacterium-targeted therapies could significantly inhibit glioma proliferation by increasing the abundance of bifidobacterium (14). In addition, the progression of glioma disrupts the intestinal mucosal barrier (30). However, bifidobacterium has not yet demonstrated the ability to repair the intestinal barrier damage caused by glioma, indicating significant potential for future research in this area. In addition, the interaction network between the gut microbiota and glioma is far more complex than currently understood. From the mechanisms by which metabolites cross the blood-brain barrier (BBB) to the precise regulation of tumor cells and the immune microenvironment, as well as the pathways influencing treatment resistance and innervation, many significant unknowns remain. Addressing these fundamental challenges will establish a critical scientific foundation for developing novel glioma prevention and treatment strategies based on microbiota regulation. Currently, there are limited studies exploring emerging topics such as the metabolites of intestinal flora and the enteric nervous system, which warrant our ongoing attention moving forward (The main microbial pathways that affect glioma are shown in **Supplementary Material 2**).

In conclusion, this study systematically describes the influence of gut microbiota on glioma progression. Through the analysis of the interaction between gut microbiota and glioma, we have gained a clearer understanding of the gut-brain axis in relation to glioma. The heme enzyme IDO-1 is a regulatory enzyme in the kynurenine pathway mediated by the intestinal microbiota. It plays a key role in tryptophan degradation and is a critical component of the gut-brain axis in neuroeducation. Kynurenine is the primary pathway for tryptophan metabolism, occurring mainly in the liver, immune cells, glial cells (astrocytes and microglia), and tumor cells. Hyperactivation of the kynurenine pathway is commonly observed in high-grade gliomas (GBM). Currently, mechanistic analyses of the midgut-brain axis in glioma primarily derive from the following studies: 1. *In vitro* experiments: Using a neuroblastoma cell line to construct a cellular model, researchers have demonstrated that overexpression of IDO-1 promotes the release of the neurotoxic metabolite quinolinic acid (QUIN) by directing kynurenine metabolism and limiting neuroprotection. This process increases tumor cell persistence and promotes pathogenesis (31). 2. Animal studies: Experiments with genetically modified mice and rats have shown that intestinal microorganisms can produce tryptophan metabolites (such as indole and its derivatives) through enzymatic transformation. These metabolites influence nervous system development and disease occurrence (32). 3. Human studies: Clinical investigations have revealed that IDO-1 is closely associated with brain cancer. The uptake and breakdown of tryptophan in glioma tissues are significantly elevated compared to normal tissues (33). Further analysis has confirmed the presence of a gut-brain axis in human glioma development. Initially, researchers observed abnormally high expression of IDO-1 in glioma cell lines, with higher expression correlating with increased tumor cell activity. IDO-1 is closely linked to tryptophan metabolism in the gut microbiota. Subsequent animal studies showed that intestinal flora activity in experimental mice significantly affects tryptophan metabolism and interferes with nervous system development via enzymatic pathways. Further research confirmed a similar mechanism in humans, verifying the potential role of intestinal flora in influencing glioma growth through IDO-1 activity. Therefore, regulating the composition of gut microbiota may represent a new direction for glioma treatment, and further in-depth and effective research is necessary in the future.

Based on the current evidence bottleneck and clinical needs, we need to further explore the therapeutic potential of microbiota modulation in glioma in the future. We can start by: (i) exploring the delivery of drug resistance genes by microbiota-derived outer membrane vesicles (OMVs) to cancer cells; (ii) decoding the code of microbial-tumor cell direct interaction using single-cell/spacomics; (iii) improving outcomes through “synthetic microbiota therapy”; (iv) establish an international glioma microbiome database and incorporate gut microbiota parameters into the precision treatment system for glioma.

### 4.6 Value and limitations of this study

Bibliometrics offers an effective method for exploring the correlation between gut microbiota and glioma. This approach enhances our understanding of the potential therapeutic value of gut microbiota in the treatment of glioma. Based on bibliometric analysis, we investigate and apply various therapies involving intestinal flora to improve the prognosis of glioma patients. Simultaneously, the preliminary bibliometric analysis provides a valuable reference for further animal experiments and human trials, thereby promoting the development of clinical drugs to some extent. In this paper, we utilized three different bibliometric tools, among which VOSviewer and CiteSpace are widely recognized for their effectiveness in bibliometric analysis, ensuring the reliability of our results. However, this study primarily focuses on analyzing English literature within the Web of Science Core Collection (WoSCC) database, which may overlook significant studies published in other languages. Limiting the analysis to articles published in English-language journals risks introducing regional and country biases. Additionally, we did not analyze other databases (e.g., Scopus, PubMed), which may have caused us to overlook important international research findings. Restricting publications to English and relying solely on a single database, particularly the Web of Science Core Collection (WoSCC), represent significant limitations inherent in bibliometric research. These constraints introduce notable biases related to region, discipline, document type, and perspective, resulting in an incomplete representation of the global knowledge landscape. Currently, bibliometric analysis is constrained by the limitations of available software, making it difficult to integrate data across multiple databases and languages. When technical capabilities mature in the future, further interdisciplinary analyses and integrations can be conducted to supplement this research. Nevertheless, the literature contained in WoSCC is extensive and frequently updated, allowing us to analyze and identify recent research hotspots effectively. Furthermore, the absence of standardized threshold parameters (such as high citation thresholds, co-occurrence frequency thresholds, cluster size thresholds, etc.) in bibliometric research presents a significant methodological challenge. This issue stems from software limitations, including clustering biases in tools like VOSviewer. However, bibliometrics, which aims to reveal structure, dynamics, and interrelationships in academic communication, is a descriptive mapping of existing literature rather than an inference about causality. Therefore, our analysis does not infer statistical characteristics through a set threshold, but rather directly describes the properties of the analyzed data set.

## 5 Conclusion and prospect

In this study, bibliometric analysis was employed to systematically examine the literature on gut microbiota and glioma within the Web of Science Core Collection (WoSCC) database. The countries that published the highest number of related articles were China and the United States, while the journal that published the most articles on this topic was research on the relationship between gut microbiota and glioma primarily focuses on the following aspects: 1. Gut microbiota regulates glioma progression by modulating immune responses; 2. Gut microbial metabolites influence glioma growth; 3. Different brain tumors exhibit distinct microbial assemblages, and analyzing gut microbiota can aid in the identification of glioma and its subtypes; 4. Gut microbiota and glioma can interact through the gut-brain axis. Further investigation into the regulation of the gut-brain axis may lead to the development of new therapies for glioma. An emerging area of research involves the regulation of glioma growth and proliferation through the enteric nervous system and amino acid metabolism. This study systematically analyzed the current research status and hotspots regarding gut microbiota in glioma, establishing a foundation for future research in this field and promoting its further advancement.

## Data Availability

The original contributions presented in the study are included in the article/**Supplementary Material**. Further inquiries can be directed to the corresponding author.

## References

[1] Ostrom QT Price M Neff C Cioffi G Waite KA Kruchko C . CBTRUS statistical report: primary brain and other central nervous system tumors diagnosed in the United States in 2014-2018. Neuro Oncol. (2021) 23:iii1–iii105. doi: 10.1093/neuonc/noab200, PMID: 34608945 PMC8491279

[2] Shah V Kochar P . Brain cancer: implication to disease, therapeutic strategies and tumor targeted drug delivery approaches. Recent Pat Anticancer Drug Discov. (2018) 13:70–85. doi: 10.2174/1574892812666171129142023, PMID: 29189177

[3] Louis DN Perry A Reifenberger G von Deimling A Figarella-Branger D Cavenee WK . The 2016 world health organization classification of tumors of the central nervous system: a summary. Acta Neuropathol. (2016) 131:803–20. doi: 10.1007/s00401-016-1545-1, PMID: 27157931

[4] Mehrian-Shai R Reichardt JKV Harris CC Toren A . The gut-brain axis, paving the way to brain cancer. Trends Cancer. (2019) 5:200–7. doi: 10.1016/j.trecan.2019.02.008, PMID: 30961828 PMC6734924

[5] Meyer C Martin-Blondel G Liblau RS . Endothelial cells and lymphatics at the interface between the immune and central nervous systems: implications for multiple sclerosis. Curr Opin Neurol. (2017) 30:222–30. doi: 10.1097/WCO.0000000000000454, PMID: 28323646

[6] Hanahan D . Hallmarks of cancer: new dimensions. Cancer Discov. (2022) 12:31–46. doi: 10.1158/2159-8290.CD-21-1059, PMID: 35022204

[7] Wu P-N Xiong S Zhong P Yang W-Q Chen M Tang T-C . Global trends in research on irritable bowel syndrome and the brain-gut axis: Bibliometrics and visualization analysis. Front Pharmacol. (2022) 13:956204. doi: 10.3389/fphar.2022.956204, PMID: 36160395 PMC9493189

[8] Lythgoe MP Mullish BH Frampton AE Krell J . Polymorphic microbes: a new emerging hallmark of cancer. Trends Microbiol. (2022) 30:1131–4. doi: 10.1016/j.tim.2022.08.004, PMID: 36058787

[9] Cryan JF O’Riordan KJ Cowan SMC Sandhu KV Bastiaanssen TFS Boehme M . The microbiota-gut-brain axis. Physiol Rev. (2019) 99:1877–2013. doi: 10.1152/physrev.00018.2018, PMID: 31460832

[10] Ratsika A Cruz Pereira JS Lynch CMK Clarke G Cryan JF . Microbiota-immune-brain interactions: A lifespan perspective. Curr Opin Neurobiol. (2023) 78:102652. doi: 10.1016/j.conb.2022.102652, PMID: 36463579

[11] D’Alessandro G Lauro C Quaglio D Ghirga F Botta B Trettel F . Neuro-signals from gut microbiota: perspectives for brain glioma. Cancers (Basel). (2021) 13. doi: 10.3390/cancers13112810, PMID: 34199968 PMC8200200

[12] Jiang H Zeng W Zhang X Pei Y Zhang H Li Y . The role of gut microbiota in patients with benign and Malignant brain tumors: a pilot study. Bioengineered. (2022) 13:7847–59. doi: 10.1080/21655979.2022.2049959, PMID: 35291914 PMC9208447

[13] Nejman D Livyatan I Fuks G Gavert N Zwang Y Geller LT . The human tumor microbiome is composed of tumor type-specific intracellular bacteria. Science. (2020) 368:973–80. doi: 10.1126/science.aay9189, PMID: 32467386 PMC7757858

[14] Fan H Wang Y Han M Wang L Li X Kuang X . Multi-omics-based investigation of Bifidobacterium’s inhibitory effect on glioma: regulation of tumor and gut microbiota, and MEK/ERK cascade. Front Microbiol. (2024) 15:1344284. doi: 10.3389/fmicb.2024.1344284, PMID: 38699473 PMC11064926

[15] D’Alessandro G Antonangeli F Marrocco F Porzia A Lauro C Santoni A . Gut microbiota alterations affect glioma growth and innate immune cells involved in tumor immunosurveillance in mice. Eur J Immunol. (2020) 50:705–11. doi: 10.1002/eji.201948354, PMID: 32034922 PMC7216943

[16] Patrizz A Dono A Zorofchian S Hines G Takayasu T Husein N . Glioma and temozolomide induced alterations in gut microbiome. Sci Rep. (2020) 10:21002. doi: 10.1038/s41598-020-77919-w, PMID: 33273497 PMC7713059

[17] Huang F Wu X . Brain neurotransmitter modulation by gut microbiota in anxiety and depression. Front Cell Dev Biol. (2021) 9:649103. doi: 10.3389/fcell.2021.649103, PMID: 33777957 PMC7991717

[18] Merzak A Koochekpour S Fillion MP Fillion G Pilkington GJ . Expression of serotonin receptors in human fetal astrocytes and glioma cell lines: a possible role in glioma cell proliferation and migration. Brain Res Mol Brain Res. (1996) 41:1–7. doi: 10.1016/0169-328X(96)00058-7, PMID: 8883928

[19] Blanchart A Fernando R Häring M Assaife-Lopes N Romanov RA Andäng M . Endogenous GAB(AA) receptor activity suppresses glioma growth. Oncogene. (2017) 36:777–86. doi: 10.1038/onc.2016.245, PMID: 27375015

[20] Naghavian R Faigle W Oldrati P Wang J Toussaint NC Qiu Y . Microbial peptides activate tumour-infiltrating lymphocytes in glioblastoma. Nature. (2023) 617:807–17. doi: 10.1038/s41586-023-06081-w, PMID: 37198490 PMC10208956

[21] Sivan A Corrales L Hubert N Williams JB Aquino-Michaels K Earley ZM . Commensal Bifidobacterium promotes antitumor immunity and facilitates anti-PD-L1 efficacy. Science. (2015) 350:1084–9. doi: 10.1126/science.aac4255, PMID: 26541606 PMC4873287

[22] Ma Q Xing C Long W Wang HY Liu Q Wang R-F . Impact of microbiota on central nervous system and neurological diseases: the gut-brain axis. J Neuroinflamm. (2019) 16:53. doi: 10.1186/s12974-019-1434-3, PMID: 30823925 PMC6397457

[23] Hung AL Garzon-Muvdi T Lim M . Biomarkers and immunotherapeutic targets in glioblastoma. World Neurosurg. (2017) 102:494–506. doi: 10.1016/j.wneu.2017.03.011, PMID: 28300714

[24] Platten M Nollen EAA Röhrig UF Fallarino F Opitz CA . Tryptophan metabolism as a common therapeutic target in cancer, neurodegeneration and beyond. Nat Rev Drug Discov. (2019) 18:379–401. doi: 10.1038/s41573-019-0016-5, PMID: 30760888

[25] Dehhaghi M Kazemi Shariat Panahi H Heng B Guillemin GJ . The gut microbiota, kynurenine pathway, and immune system interaction in the development of brain cancer. Front Cell Dev Biol. (2020) 8:562812. doi: 10.3389/fcell.2020.562812, PMID: 33330446 PMC7710763

[26] Dehhaghi M Kazemi Shariat Panahi H Guillemin GJ . Microorganisms’ Footprint in neurodegenerative diseases. Front Cell Neurosci. (2018) 12:466. doi: 10.3389/fncel.2018.00466, PMID: 30564101 PMC6288487

[27] Dehhaghi M Kazemi Shariat Panahi H Guillemin GJ . Microorganisms, tryptophan metabolism, and kynurenine pathway: A complex interconnected loop influencing human health status. Int J Tryptophan Res. (2019) 12:1178646919852996. doi: 10.1177/1178646919852996, PMID: 31258331 PMC6585246

[28] Wong SH Kwong TNY Wu C-Y Yu J . Clinical applications of gut microbiota in cancer biology. Semin Cancer Biol. (2019) 55:28–36. doi: 10.1016/j.semcancer.2018.05.003, PMID: 29782923

[29] Yang Q Wang B Zheng Q Li H Meng X Zhou F . A review of gut microbiota-derived metabolites in tumor progression and cancer therapy. Adv Sci (Weinh). (2023) 10:e2207366. doi: 10.1002/advs.202207366, PMID: 36951547 PMC10214247

[30] Wang L Li S Fan H Han M Xie J Du J . Bifidobacterium lactis combined with Lactobacillus plantarum inhibit glioma growth in mice through modulating PI3K/AKT pathway and gut microbiota. Front Microbiol. (2022) 13:986837. doi: 10.3389/fmicb.2022.986837, PMID: 36147842 PMC9486703

[31] Adams S Braidy N Bessede A Brew BJ Grant R Teo C . The kynurenine pathway in brain tumor pathogenesis. Cancer Res. (2012) 72:5649–57. doi: 10.1158/0008-5472.CAN-12-0549, PMID: 23144293

[32] Zhang LS Davies SS . Microbial metabolism of dietary components to bioactive metabolites: opportunities for new therapeutic interventions. Genome Med. (2016) 8:46. doi: 10.1186/s13073-016-0296-x, PMID: 27102537 PMC4840492

[33] Zhai L Spranger S Binder DC Gritsina G Lauing KL Giles FJ . Molecular pathways: targeting IDO1 and other tryptophan dioxygenases for cancer immunotherapy. Clin Cancer Res. (2015) 21:5427–33. doi: 10.1158/1078-0432.CCR-15-0420, PMID: 26519060 PMC4681601

