# Supplementary figures and images for: A scientometric analysis of global research on gut microbiota and glioma
Source: Front Oncol. 2025 Oct 7;15:1646187. doi: 10.3389/fonc.2025.1646187 (PMC12537380; doi:10.3389/fonc.2025.1646187)

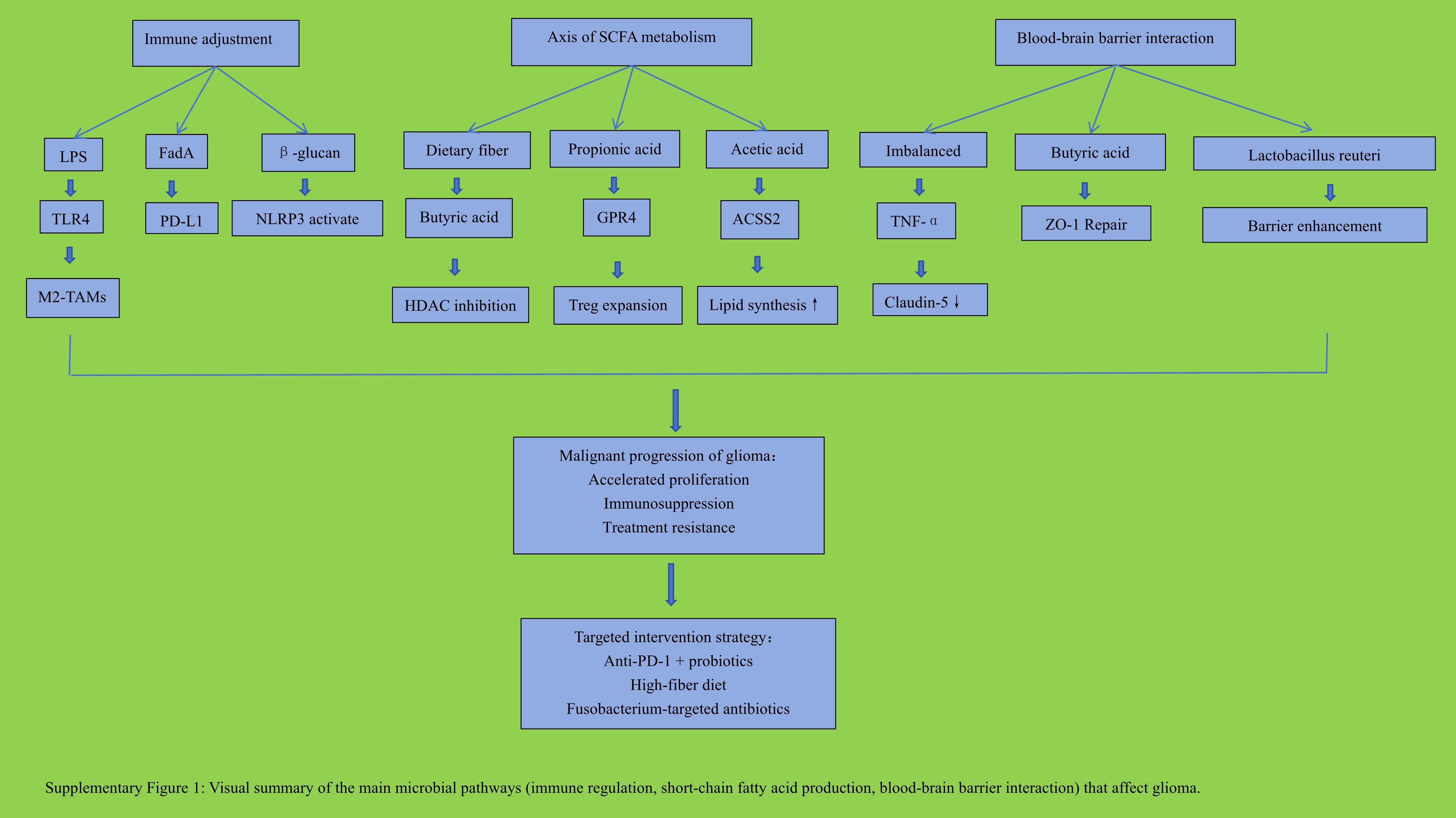

Supplement: Supplementary file 1 [file Image1.jpeg]
