# Supplementary material for: A scientometric analysis of global research on gut microbiota and glioma
Source: Front Oncol. 2025 Oct 7;15:1646187. doi: 10.3389/fonc.2025.1646187 (PMC12537380; doi:10.3389/fonc.2025.1646187)
Supplement: Supplementary file 2 [file Table1.pdf]

Year	Title of Article	Number of citations	Main contents
2020	The human tumor microbiome is composed of tumor type-specific intracellular bacteria.	1184	The composition of tumor microbes influences tumor immunity and response to immunotherapy.
2019	Impact of microbiota on central nervous system and neurological diseases: the gut-brain axis.	464	Both neural and immune activity in the brain can be determined directly by microbial metabolites or indirectly by microbiota-derived whole-body signals.
2022	Microbiota and the gut-brain-axis: Implications for new therapeutic design in the CNS.	141	Microorganisms can contribute to the production of essential metabolites, neurotransmitters, and other neuroactive compounds, thereby affecting the progression or treatment of various central nervous system diseases.

Supplementary Table 1 Top 3 articles and their main contents in co-citation.
