# Supplementary material for: A scientometric analysis of global research on gut microbiota and glioma
Source: Front Oncol. 2025 Oct 7;15:1646187. doi: 10.3389/fonc.2025.1646187 (PMC12537380; doi:10.3389/fonc.2025.1646187)
Supplement: Supplementary file 3 [file DataSheet1.pdf]

**Search strategy**

The literature collected in this study was from the Web of Science Core Collection (wosCC). The search formula was TS=(("gut microbio " OR "intestinal microbio " OR "gut flora" OR "intestinal flora" OR "gut bacteri " OR "intestinal bacteri " OR "gut microbiota" OR "gut microbiome" OR "microbiota-gut-brain" OR "microbiome-gut-brain" OR "gut-brain axis" OR "brain-gut axis" OR "enteric nervous system" OR "gastrointestinal microbio " OR "fecal microbio " OR "gut dysbios " OR "intestinal dysbios " ) AND (glioma OR glioblastoma OR "brain tumor " OR "brain tumour " OR "brain neoplasm " OR astrocytoma OR oligodendroglioma OR "neuroepithelial tumor " OR "neuroepithelial tumour " OR "GBM" OR "grade IV glioma" OR "high-grade glioma" OR "low-grade glioma" OR "diffuse intrinsic pontine glioma" OR "DIPG" OR "IDH-mutant" OR "IDH-wildtype")), the article types were set to "article" and "review article", the language was limited to "English", and the search date was from January 1, 2005 to April 11, 2025. According to our search strategy, a total of 127 documents were retrieved, among which 43 were reviews and 84 were articles.

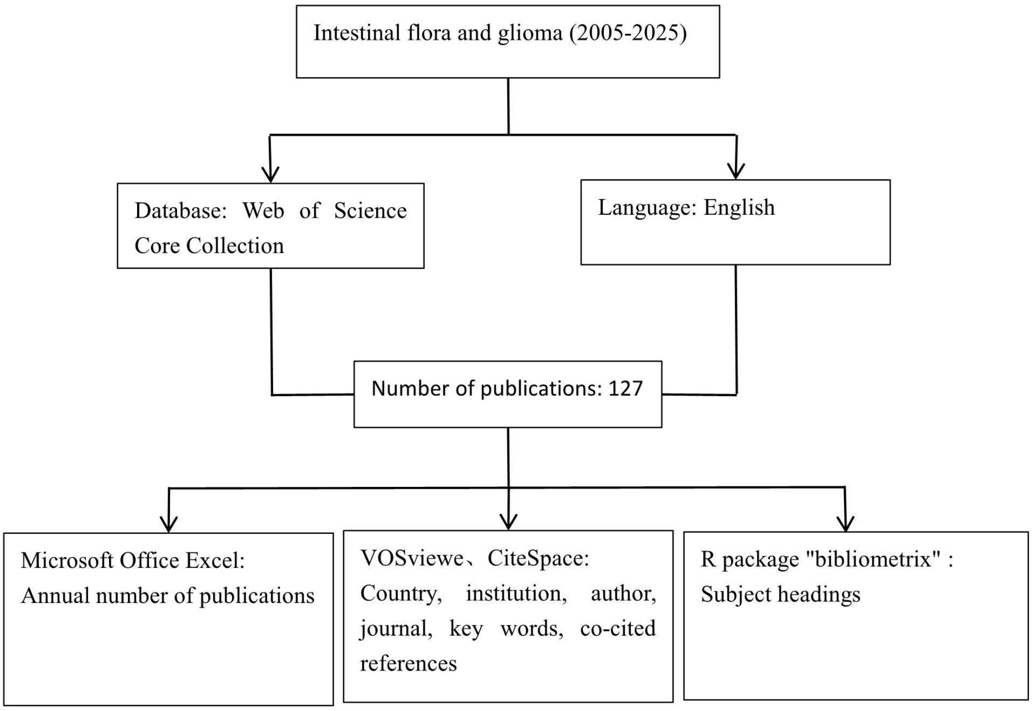


Figure 1. Research flowchart.
